# HA380 Hemoperfusion Combined with Continuous Veno-Venous Hemodiafiltration for the Treatment of Septic Shock

**DOI:** 10.3390/bioengineering12040400

**Published:** 2025-04-09

**Authors:** Yuling An, Yi Guo, Wenjuan Zhou, Qinqin He, Ziyu Li, Xin Sui, Xiaomeng Yi, Huimin Yi

**Affiliations:** 1 Department of Surgical Intensive Care Unit (SICU), Third Affiliated Hospital of Sun Yat-sen University, Guangzhou 510630, China; zhouwenjuan19870@163.com (W.Z.); heqq8@mail.sysu.edu.cn (Q.H.); lizy225@mail.sysu.edu.cn (Z.L.); drsuixin@126.com (X.S.); yixiaom@mail.sysu.edu.cn (X.Y.); 2 Department of Surgical Intensive Care Unit (SICU), Fuwai Yunnan Cardiovascular Hospital, No.528, Shahe North Road, Kunming 650021, China; yizi82@icloud.com

**Keywords:** hemoperfusion, continuous veno-venous hemodiafiltration, septic shock, sepsis, inflammatory factors, prognosis

## Abstract

Objective: To explore the synergistic effect of HA380 hemoperfusion adsorbent combined with continuous veno-venous hemodiafiltration (CVVHDF) in the treatment of septic shock. Patients and methods: This retrospective study included 56 adult septic shock patients who underwent blood purification treatment in the Surgical Intensive Care Unit (SICU) of Third Affiliated Hospital of Sun Yat-sen University from 1 September 2022 to 1 April 2024. Twenty patients received a combination therapy of HA380 hemoperfusion and CVVHDF, while 36 patients received only CVVHDF treatment. Inflammatory markers such as white blood cells (WBC), procalcitonin (PCT), and interleukin-6 (IL-6) were evaluated. Changes in acute physiology and chronic health status evaluation system (APACHE II) scores, sequential organ failure (SOFA) scores, and outcomes at 7 and 28 days after treatment were recorded. Result: After 24 h of treatment, the levels of PCT and IL-6 in the combined group decreased significantly (*p* < 0.05). The 7-day and 28-day mortality rates of the combined group were 25% and 30%, respectively, while the mortality rates of the CVVHDF group were 27.8% and 50%, respectively. Conclusion: HA380 hemoperfusion combined with CVVHDF was safe and effective in treating patients with septic shock.

## 1. Introduction

Septic shock is a common clinical syndrome in the field of critical care medicine, often accompanied by multiple organ dysfunction and insufficient tissue perfusion, with a high mortality rate [1,2]. Blood purification is a commonly used treatment to inhibit the progression of sepsis, although its effectiveness in improving prognosis remains controversial [3,4].

Continuous veno-venous hemodiafiltration (CVVHDF) is one of the methods of continuous kidney replacement therapy (CRRT), which is used to treat patients with septic AKI whose renal function is no longer preserved and can reduce their mortality [5]. CVVHDF has been shown to be effective in eliminating toxins and lowering cytokine levels in patients [6]. However, due to acute phase response, self-hydrophilicity, and molecular weight limitations, the clearance rate of CVVHDF is limited. CVVHDF can only remove small or medium molecular substances, and its clearance of large molecular inflammatory mediators is limited. Hemoperfusion (HP) can play a good role in removing large molecular inflammatory mediators [7,8]. However, hemoperfusion is less effective than CVVHDF in regulating electrolyte and acid-base balance [5,7]. Therefore, one of the research hotspots has been how to optimize the use of hemoperfusion combined with blood filtration [9,10].

This study aimed to explore the synergistic effect of hemoperfusion and CVVHDF in the treatment of septic shock based on inflammatory response, coagulation function, and end-organ damage markers in order to provide a reference for clinical treatment.

## 2. Patients and Methods

### 2.1. General Information

We conducted a retrospective study to explore the therapeutic efficacy of HA380 hemoperfusion combined with continuous blood purification for adult septic shock patients admitted to the SICU of the Third Affiliated Hospital of Sun Yat-Sen University from September 2022 to April 2024. The study was approved by the Ethics Committee of the Third Affiliated Hospital of Sun Yat-Sen University for the analysis of the data from the database and conducted in accordance with the Helsinki Declaration of 1975. The inclusion criteria included patients diagnosed with septic shock with acute kidney injury (AKI) within 72 h and had classical indications for a substitutive treatment of renal function (fluid overload, hyperkalemia, acidosis, and so on), age range of 19–75 years, Acute Physiology and Chronic Health Evaluation II (APACHE II) score of patients ≥15 points, Sequential Organ Failure Assessment (SOFA) score ≥2 points, and received bedside CVVHDF treatment in the ICU for more than 24 h. The exclusion criteria included age over 75 years old, active bleeding, thrombocytopenia (platelet count below 50 × 10^9^/L), late stage of malignant tumors, immunodeficiency diseases, organ transplantation, HIV infection, and refusal or failure to perform CRRT for 24 h.

AKI was defined by the 2012 KDIGO AKI guideline [11]. Septic shock was defined as the need for vasopressors to maintain mean arterial pressure ≥65 mmHg and serum lactate levels above 2 mmol/L despite sufficient fluid therapy, according to the third international consensus definition of sepsis and septic shock [1]. Disease severity and organ function were assessed using the APACHE *II* score and SOFA score to predict mortality in general ICU patients [12].

### 2.2. Blood Purification Therapy

All septic shock patients received basic treatments such as fluid resuscitation, anti-infective agents, vasopressors, and mechanical ventilation. A double-lumen catheter was established for vascular access in the internal jugular or femoral vein by using the Seldinger technique. The Aquarius V6 acute dialysis and extracorporeal blood therapy machine (Shanghai Nikkiso Co., Ltd., Shanghai, China) was used for blood purification treatment. Diacap Acute M-type polysulfone membrane filters (B. Braun Avitum Saxonia GmbH, Melsungen, Germany) and CVVHDF treatment mode were chosen. All the blood purification therapy was carried out according to the company’s user manual. Bicarbonate replacement solution and citrate anticoagulant were used for anticoagulation. The blood flow rate was set to 150–200 mL/min, and the total daily dialysis dose of CVVHDF is 35 mL/kg/h (pre-dilution and postdilution 1:1 mode). In the combined hemoperfusion (HP) and CVVHDF treatment group, concurrent CVVHDF and hemoperfusion were performed. Hemoperfusion was undertaken with a resin cartridge (HA-380, Jafron Biomedical, Zhuhai, China) once a day. The hemoperfusion cartridge preceded the hemofilter in the circuit. Each HA380 hemoperfusion session lasted from 3.5 to 5 h. The HA380 hemoperfusion cartridge was removed from the pipeline after the completion of hemoperfusion treatment and continued CVVHDF treatment. In the control group, only CRRT with CVVHDF mode was performed at least 24 h every time.

Before and after each blood purification treatment, data on inflammatory markers [white blood cells (WBC), procalcitonin (PCT), interleukin-6 (IL-6)], and organ dysfunction markers, including APACHE *II* score, SOFA score, platelet count, alanine aminotransferase (ALT), aspartate aminotransferase (AST) levels, and creatinine levels were recorded. Mortality was observed 7 days and 28 days after treatment in both groups.

### 2.3. Statistical Analysis

SPSS 25.0 statistical software (IBM Corporation, Chicago, IL, USA) was used for data analysis. Data was expressed as mean ± standard deviation (SD). The Kolmogorov–Smirnov test was used to check if the data were normally distributed. Wilcoxon signed-rank test or *t*-test was used for comparison. The Chi-square test was used to compare the rates of the two groups. Survival analysis was conducted using Kaplan–Meier curves, which were tested with a log-rank test, while hazard ratios were estimated with Cox proportional hazards regression. Binary logictic regression was used to explore the impact of different variables on ICU survival. We constructed a regression model that included the APACHE II score, comorbidities, COVID-19 infection, CRRT mode, and other factors to assess the independent associations of multiple independent variables with patients’ survival. A *p*-value < 0.05 was considered to be statistically significant.

## 3. Results

### 3.1. Baseline Characteristics and Outcome

All septic shock patients received mechanical ventilation in the ICU. The baseline demographic and clinical characteristics of the patients were summarized in Table 1. The patients’ ages ranged from 39 to 72 years old, with a median of 57 years old. Based on clinical manifestations and cultivation data, septic shock was caused by respiratory infection in 21 patients, intra-abdominal infection in 12 patients, urinary tract infection in 8 patients, skin and soft tissue infection in 6 patients, blood stream infection in 4 patients, and unknown infection in 5 patients.

Twenty patients received a median of 2 sessions and a total of 33 sessions of HA380 hemoperfusion. The mortality rates of the combined HP and CVVHDF group after 7 and 28 days of treatment were 25% and 30%, respectively, while the mortality rates of the CVVHDF group after 7 and 28 days of treatment were 27.8% and 50%, respectively. The patients were followed for 28 days; 14 patients in the combined HP and CVVHDF group and 18 patients in the CVVHDF patients survived.

**Figure 1:**
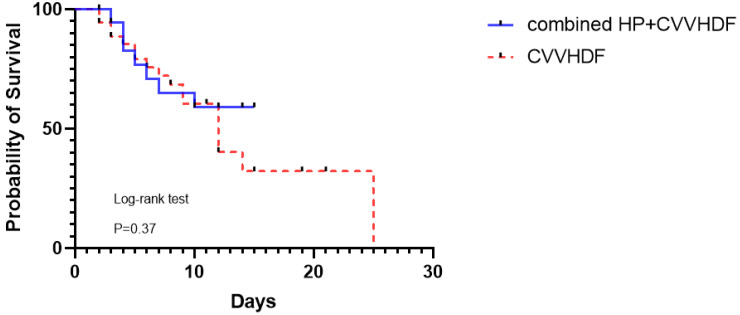
Outcome for 28-day survival in ICU all patients subdivided by combined HP + CVVHDF or CVVHDF treatment. at the end of follow-up.

From the regression model, we did not find any patient or disease factors that affected the survival rate of enrolled patients (data shown in Table 2). We did not find a significant difference in survival between the two groups at the end of the follow-up period (Figure 1).

### 3.2. The Impact of Blood Purification on Indicators of Organ Dysfunction

Some organ function indicators were analyzed before and 24 h after blood purification treatment. Before treatment, there were no significant differences in organ indicators between the two groups. After 24 h of blood purification treatment, the lactate, creatinine, ALT, AST, and platelet counts in both groups were significantly decreased compared to before treatment (*p* < 0.05). Although there was no significant change in SOFA scores in both groups, the APACHE II score in the combined HP and CVVHDF group was significantly decreased compared to the CVVHDF group. (Data shown in Table 3).

### 3.3. The Change of Inflammatory Markers

Before treatment, there were no significant differences in inflammatory markers between the two groups. After 24 h of treatment, the levels of WBC and lactic acid in both groups of patients decreased significantly, especially in the combined HP and CVVHDF group (*p* < 0.05). The levels of PCT and IL-6 in the combined HP and CVVHDF group decreased significantly (*p* < 0.05), while the decrease was not significant in the CVVHDF group (*p* > 0.05) (data shown in Table 3).

## 4. Discussion

Sepsis is characterized by systemic inflammatory response syndrome (SIRS), which can lead to multiple organ failure and death. The mortality rate of hospital sepsis is as high as 44%, and the mortality rate of septic shock can increase to 59% [1]. In order to provide better treatment for patients with septic shock, we actively explore the optimization mode of blood purification therapy. We analyzed the treatment plan of hemoperfusion combined with CVVHDF and found that compared with CVVHDF alone, hemoperfusion can clear inflammatory factors more quickly and protect organ function.

Inflammation imbalance and immune function abnormalities are considered important causes of death in sepsis patients [13]. Removing endotoxins and plasma cytokines through an extracorporeal blood purification device can control related immune system dysregulation and may be associated with improved prognosis in sepsis patients [13,14]. Renal replacement therapy (RRT) technology can be employed to use specially developed adsorption membranes, blood perfusion adsorbent kits, or in vitro column adsorption of inflammatory mediators. These technologies aim to reduce the levels of circulating proinflammatory cytokines and endotoxins in order to improve sepsis patient prognosis [15]. CVVHDF and hemoperfusion are commonly used blood purification techniques for sepsis treatment by removing inflammatory cytokines from the blood [16]. Especially, blood adsorption technology represented by hemoperfusion is a potential and useful treatment method, as its molecular weight clearance range is wide and can reduce the peak levels of endotoxins and cytokines [17]. Erkurt et al. used the HA330 hemoperfusion adsorbent method as a last resort in terminal sepsis patients with a mortality rate of approximately 90% [8]. Kaçar et al. conducted a prospective study on the results of HA330 blood perfusion for 23 sepsis patients for 3 days and 2 h per day and found that CRP and procalcitonin levels significantly decreased after the second application [18]. However, blood perfusion alone does not seem to improve the overall survival of sepsis patients. In combination with the different transport mechanisms, a number of RRT modalities are identified and described, using parallel, series, or sequential methods to leverage their respective advantages and achieve complementary advantages [7,14].

Applying blood perfusion combined with blood filtration in sepsis is an emerging treatment method aimed at clearing inflammatory mediators and harmful substances in the blood, thereby alleviating the condition of sepsis patients [7,9]. Zheng et al. conducted a study that the use of hemoperfusion (Adsorba 300) combined with CRRT treatment significantly improved the 30-day survival rate in early sepsis patients [19]. The HA380 cartridge is a new type of adsorption hemoperfusion device, which is suitable for acute inflammatory diseases such as sepsis, trauma, burn, pancreatitis, and various cytokine release syndromes (such as severe COVID-19) [20,21, 22,23]. In vitro testing suggested it had the best level of biocompatibility and was not associated with adverse reactions or signs of cytotoxicity [20]. No adverse events, such as thrombocytopenia, were observed in the HA380 treatment group [23].

This study investigated the clinical efficacy of using the latest disposable hemoperfusion device, HA380, in series with CVVHDF in the treatment of septic shock. Preliminary results were convincing, showing that HA380 combined CVVHDF had advantages in eliminating inflammatory mediators, reducing infection symptoms, and promoting organ function recovery.

In this study, the levels of WBC, IL-6, and procalcitonin (PCT) were significantly decreased after HA380 hemoperfusion combined with CVVHDF. Procalcitonin is a precursor of calcitonin, which is abnormally elevated in patients with sepsis. Monitoring changes in PCT can predict the prognosis of sepsis and septic shock [24,25]. Interleukin-6 (IL-6) is a pro-inflammatory cytokine produced in fibroblasts, endothelial cells, T lymphocytes, and monocytes. It is an important cellular mediator in the acute inflammatory phase of sepsis, indicating the severity of sepsis [25,26]. IL-6 with a large molecular weight can serve as the main factor for evaluating the prognosis of sepsis. The decrease of IL-6 after sepsis treatment may be an indicator of better prognosis and survival of patients with sepsis [25,26]. Although blood lactate is not an inflammatory mediator, it can help identify occult shock and has important value in predicting the prognosis of sepsis [4]. The decrease in these inflammatory factors was mainly due to the filtration effect of CVVHDF combined with the adsorption effect of HA380 blood perfusion.

Some organ dysfunction markers were also evaluated in this study. After 24 h of treatment in the combination therapy group, lactate, creatinine, ALT, and APACHE II scores significantly decreased compared to before treatment, indicating that this combination therapy can significantly improve organ function. However, we did not observe sufficient clinical improvement in these two groups of septic shock patients, and there was no significant difference in mortality rates between the two groups at 7 and 28 days. In addition, a slight decrease was noted in platelet count in these patients, which was similar to the reported side effects of other blood perfusion or adsorption therapies [27,28,29].

At present, cytokine adsorption technology, represented by hemoperfusion, is a promising technology for the treatment of septic shock, and it has a remarkable effect in patients with specific shock, such as burns [30]. However, large trials with null results suffered from the presence of many potential confounding factors, such as the use in unselected patient populations, variability in demographic characteristics, comorbidities, use of medication, time since disease onset, source of the infection, or duration of therapy. It is undeniable that our study has many confounding factors affecting survival rates. There were also some differences in baseline characteristics, such as age, between the two groups. It is particularly important to state that the treatment of sepsis is a complex process, and it is difficult to improve survival through a single treatment mode [15]. Dealing with the primary lesion of sepsis is also particularly important, especially for sepsis patients caused by trauma, which requires surgical intervention such as infection tissue clearance, abscess drainage, and/or resection [4]. More clinical research and practice are still needed to further verify the efficacy and safety of this blood purification therapy and provide more guidance and experience for clinical application.

## 5. Limitations

The main limitations of our study are its retrospective design, small sample size, short total treatment time for hemoperfusion, and the selection of patient group from a high-mortality group with a heterogeneous background. Prospective and randomized studies are needed on more patients to elucidate the effectiveness of this combined treatment modality for septic shock.

## 6. Conclusions

This study preliminarily confirmed that HA380 hemoperfusion combined with CVVHDF, a novel RRT modality, was safe and effective in treating patients with septic shock.

## Figures and Tables

**Table 1:** Baseline characteristics and outcome of patients (*n* = 56).

Variable	The Combined HP and CVVHDF Group (*n* = 20)	The CVVHDF Group (*n* = 36)	*p*-Value
Age (years) mean ± SD	54 ±7.9	59.6 ± 6.6	0.0063
Gender (Female/male), n	6/14	8/28	0.52
Body mass index (kg/m^2^) mean ± SD	23.24 ± 2.04	24.01 ± 2.44	0.23
Comorbidities before hospitalization			0.74
Hypertension (yes) N (%)	4 (20%)	6 (16.7%)	
Diabetes (yes) N (%)	2 (10%)	4 (11.1%)	
Organ transplantation (yes) N (%)	1 (5%)	2 (5.6%)	
Leukemia (yes) N (%)	1 (5%)	0	
Surgery during hospitalization (yes) N (%)	18 (90%)	35 (97.2%)	0.25
Duration of ICU (days)	10 (2–15)	8 (2–25)	0.74
Duration of hospitalization (days)	20 (9–28)	18 (6–34)	0.16
Daily Blood pump flow (mL/h)	150 (150–180)	150 (150–180)	0.95
Daily UFR (mL/h)	110 (60–140)	105 (60–150)	0.64
Daily fluid balance (mL)	175 (−1000–600)	125 (−1200–700)	0.65
Infection site, n (%)			0.08
Respiratory infection	9 (45%)	12 (33.3%)	
Intra-abdominal infection	4 (20%)	8 (22.2%)	
Urinary tract infection	1 (5%)	7 (19.4%)	
Skin and soft tissue infection	2 (10%)	4 (11.1%)	
Blood stream infection	1 (5%)	3 (8.3%)	
Other or unknown infection	3 (15%)	2 (5.6%)	
Pathogen n (%)			0.07
Gram-positive bacteria	2 (10%)	4 (11.1%)	
Gram-negative bacteria	8 (40%)	14 (38.9%)	
COVID-19 virus	3 (15%)	6 (16.7%)	
Other viruses	1 (5%)	2 (5.6%)	
Mixed	3 (15%)	8 (22.2%)	
Unknown	3 (15%)	2 (5.6%)	
Mechanical ventilation, (n) %	20 (100%)	36 (100%)	1
APACHE II	18.40 ± 2.58	19.86 ± 3.37	0.22
SOFA	16 (14–17)	16 (13–18)	0.82
Lactic acid (mmol/L)	4.24 ± 1.52	5.13 ± 1.15	0.34
7 days mortality	5 (25%)	10 (27.8%)	0.82
28 days mortality	6 (30%)	18 (50%)	0.15

HP = hemoperfusion, CVVHDF = continuous veno-venous hemofiltration, UFR = ultrafiltration rate, APACHE = acute physiology and chronic health evaluation, SOFA = sequential organ failure assessment.

**Table 2:** Binary logictic regression model.

		B	S.E.	Wald	df	Sig.	Exp (B)
Step 1 (a)	Age	0.032	0.061	0.274	1	0.601	1.032
	BMI	−0.054	0.136	0.161	1	0.688	0.947
	APACHE II	1.591	0.842	3.569	1	0.059	4.910
	Comorbidity	−0.328	1.491	0.048	1	0.826	0.720
	COVID-19 infection	−1.201	0.958	1.572	1	0.210	0.301
	CRRT mode	−0.360	0.322	1.248	1	0.264	0.698
	Lactic acid	0.001	0.024	0.001	1	0.974	1.001
	PCT	−0.002	0.001	2.334	1	0.127	0.998
	interleukin-6	−0.055	0.138	0.158	1	0.691	0.947
	Constant	3.466	5.758	0.362	1	0.547	32.006

a Variable(s) entered on step 1: age, BMI, APACHE II, COVID-19 infection, CRRT mode, Lactic acid, PCT, interleukin-6.

**Table 3:** Laboratory and physiological variables before and after 24 h in two groups [mean ± SD/median (IQR)].

Group	The Combined HP and CVVHDF Group (n = 20)		The CVVHDF Group (n = 36)	
	0 H (Before Treatment)	24 H (After 24 h Treatment)	0 H (Before Treatment)	24 H (After 24 h Treatment)
PLT (10^9^/L)	83.80 ± 45.06	74.40 ± 36.01 *	92.92 ± 29.16	84.86 ± 25.38 *
Creatinine (μmol/L)	166.6 ± 88.9	99.25 ± 19.95 *#	223.0 ± 100.2	143.6 ± 52.46 *
ALT (U/L)	102.5 ± 54.14	83.20 ± 33.56 *#	154.1 ± 80.14	138.9 ± 72.79 *#
AST (U/L)	301.1 ± 539.1	190.0 ± 323.5 *	197.4 ± 265.8	170.5 ± 221.5 *
PT (s)	21.34 ± 1.74	20.8 ± 2.03	24.37 ± 2.90	24.18 ± 3.14
APACHE II	18.40 ± 2.58	14.25 ± 2.53 *#	19.86 ± 3.37	18.81 ± 3.42
SOFA	16 (14–17)	16 (14–17)	16 (13–18)	16 (10–18)
WBC (109/L)	17.36 ± 6.78	15.02 ± 3.88 *#	19.64 ± 6.58	18.56 ± 6.24 *
interleukin-6 (mg/L)	335.4 (94.62–5000)	249.4 (17.30–1978) *	355 (156.4–5000)	362.3 (132.2–3800)
PCT (mg/L)	21.46 ± 26.01	15.84 ± 17.70 *	19.39 ± 21.79	17.37 ± 16.13
Lactic acid (mmol/L)	4.24 ± 1.52	2.63 ± 0.70 * #	5.13 ± 1.15	4.09 ± 0.86 * #

CVVHDF = continuous veno-venous hemodialysis filtration, PLT = platelet, ALT = alanine transaminase, AST = aspartate transaminase, PT = prothrombin time, APACHE = acute physiology and chronic health evaluation, SOFA = sequential organ failure assessment, WBC = white blood cell count, PCT = procalcitonin. Lac = lactic acid. * *p* < 0.05, vs. 0 H in the same group; # *p* < 0.05, vs. 24 H in different group.

## Data Availability

All data generated or analyzed during this study were included in this published article.
